# Sodium and potassium analysis of individual coccoliths by secondary ion mass spectrometry

**DOI:** 10.1038/s41598-026-40623-2

**Published:** 2026-02-28

**Authors:** Anne Roepert, Jack J. Middelburg, Gabriella M. Weiss, Marcel T. J. van der Meer, Lubos Polerecky

**Affiliations:** 1 Department of Earth Sciences, Utrecht University, PO Box 80021, 3508 TA Utrecht, The Netherlands; 2 Soil Chemistry, Department of Environmental Sciences, Wageningen University, PO Box 47, 6700 AA Wageningen, The Netherlands; 3 Department of Marine Microbiology and Biogeochemistry, NIOZ Royal Netherlands Institute for Sea Research, 1790 AB Den Burg, The Netherlands; 4 Center for Space Science and Technology, University of Maryland Baltimore County, Baltimore, MD USA; 5 Center for Research and Exploration in Space Science and Technology II, NASA/GSFC, Greenbelt, MD USA

**Keywords:** Coccolithophores, *Gephyrocapsa huxleyi* (*Emiliania huxleyi*), Environmental proxies, Elemental ratios, NanoSIMS, Palaeoceanography, Palaeoclimate

## Abstract

Coccoliths are individual plates of calcium carbonate that comprise the shells of marine calcifying haptophyte algae. Their remains provide an excellent sedimentary archive for the reconstruction of past environmental parameters. Using nano-scale secondary ion mass spectrometry (NanoSIMS), we measured the Mg, Sr, Na and K contents in individual coccoliths of *Emiliania huxleyi*, now named *Gephyrocapsa huxleyi,* to explore their potential as a paleoproxy. For recent environmental samples from the Mediterranean and Black Sea as well as cultured specimens, all elements appeared to be homogeneously distributed within, but highly variable among, the individual coccoliths. Mg/Ca and Sr/Ca ratios covered a range of 0*.*06–83 mmol mol^−1^ and 1*.*9–3*.*8 mmol mol^−1^, respectively, and were in line with the previously determined values. Na/Ca ranged between 1*.*6–186 mmol mol^−1^. K/Ca could not be calibrated, but the measured ^39^K^+^/^44^Ca^+^ ion count ratios varied between 0*.*03–2*.*7. Although the Na/Ca ratios significantly decreased with increasing total alkalinity and salinity in the Mediterranean samples, these trends were not observed in samples collected from cultures where the alkalinity and salinity varied separately. Similarly, K/Ca ratios showed no clear trends with total alkalinity or salinity of the culture medium. Calcification in coccolithophores is biologically controlled and this may mask the impact of environmental factors on the observed variation in the Na and K content of individual coccoliths of *E.* *huxleyi*.

**Supplementary Information:**

The online version contains supplementary material available at 10.1038/s41598-026-40623-2.

## Introduction

Coccolithophores are ubiquitous marine haptophyte algae that evolved in the Upper Triassic^1^. They play a crucial role in the carbon cycle^2,3^, and because their remains (both organic and inorganic) are preserved in many sedimentary records, they provide an excellent archive for the reconstruction of past environmental parameters.

Traditional approaches of paleo-environmental reconstruction involving coccolithophores use species assemblages to reconstruct regional climate based on growth limits of different species (e.g., Ref.^4,5^), or the coccolith size distributions to estimate carbonate system parameters (e.g., Ref.^6^). Other approaches are based on organic proxies such as biomarkers specific to haptophyte algae. Of these, alkenones are the most studied compounds^7^. For example, the U^K^_37_ index was one of the first organic temperature proxies developed^8,9^. It is based on the relative extent of unsaturation in alkenones of haptophyte algae and has been shown to be related to average annual mean sea surface temperature^8,10^. Other alkenone-derived proxies are based on their isotopic signatures, e.g., the carbon isotopic composition can be used to reconstruct past *p*CO_2_ levels^11,12^, and the hydrogen isotopic composition has been shown to relate to growth water hydrogen isotope values and salinity^13,14^.

Coccoliths, which are individual plates of calcium carbonate that comprise the shells of coccolithophores, have a great potential as carriers of inorganic proxies. However, due to their small size, they have been understudied compared to the shells and skeletons of other marine calcifiers such as foraminifera or corals. Picking individual coccoliths is a tedious task, and there is high risk of contamination due to the high surface/volume ratio of coccoliths, implying the need for labour-intensive cleaning procedures. Nevertheless, numerous attempts have been made to test and develop proxies based on coccolith calcite, often preceded by those based on foraminiferal calcite. The range of element and stable isotope ratios that have been investigated to date include Sr/Ca^15–19^, Mg/Ca^17,20^, B/Ca^21^, Ba/Ca^22^, Zn/Ca^23^, *δ*^13^C^24^, *δ*^18^O^25,26^, *δ*^26/24^ Mg (e.g., Ref.^20,27^), *δ*^44/40^Ca (e.g., Ref.^27,28^), δ^88/86^Sr^29,30^, and clumped oxygen isotopes^31^. Analytically, studies of the inorganic geochemistry of coccoliths progressed from bulk chemical methods applied to coccolith rich sediments like coccolith oozes^32^, coccolith size fractions of marine sediments^33–35^, and coccoliths obtained from culture pellets of various species^15,24,26,30,36,37^, towards investigations of the chemical composition of initially a few and later individual coccoliths and/or coccolithophorid cells on a species-specific level with high spatial resolution techniques such as secondary ion mass spectrometry (SIMS)^19,21,38^, nano-scale (Nano)-SIMS^39–41^, time of flight (TOF)-SIMS^42^, synchrotron X-ray imaging^43–46^, and scanning transmission electron microscopy coupled to energy dispersive X-ray spectroscopy (STEM-EDX^46^).

Sodium has been proposed as a candidate for paleo-salinity reconstructions from foraminiferal calcite^47–51^. Sodium is a major, conservative element in seawater and can be incorporated in the interstitial sites of the calcite lattice or associated with intra-shell organic material in foraminifera^49^. To the best of our knowledge, the potential of Na content of coccoliths as a proxy for salinity has not yet been explored. Recently, Nambiar et al.^52^ explored potassium incorporation into foraminifera and other calcifiers and observed that K incorporation is rather independent of seawater and environmental parameters. Both Na and K were found in the Ca-rich body and Ca-P-diluted coccolith precursor phases inside cells of *E.* *huxleyi* (recently renamed to *G. huxleyi*^53,54^), while no measurable amounts of Na and K were detected in coccolith calcite within the calcification vesicle^46^. In partly overgrown Middle Jurassic *Watznaueria britannica* heterococcoliths, K/Ca ratios in the range of 0*.*1–24*.*9 mmol mol^−1^ were found using synchrotron X-ray fluorescence imaging^45, while^ Na/Ca was not measured. Calcification in foraminifera and coccolithophores involves different mechanisms, and it is therefore instructive to explore the Na and K content in coccoliths for their proxy potential.

Compared with the commonly determined minor and trace elements in coccoliths, determination of Na and K faces additional analytical challenges due to potential contamination with sea salts. Na and K are major conservative constituents of seawater and the coccolith Na and K content is expected to be low. Furthermore, due to the high surface/volume ratio linked to the small size and distinct geometry of the coccoliths, surface adsorption or precipitation of sea salts onto the coccolith surface is a major challenge for the chemical analyses of coccolith calcite. In addition to sea salt, contamination with trace elements of non-carbonate sources like organics is an issue, as has been shown for Mg^55–57^. Since cytosolic as well as organic-bound Na and K would likely precipitate as salts or stay bound to organic cell material during sample preparation, chemical cleaning needs to be applied to remove salts and organics. Most cleaning protocols, however, have originally been developed for foraminifera and can be sub-optimal for coccolith calcite due to extensive dissolution. Furthermore, many of the chemicals typically used in coccolith cleaning procedures are Na-based, like sodium hypochlorite or mixtures containing sodium hydroxide^55^, or alternatively K-based (respective compounds). Hence, the potential for contamination during chemical cleaning is high.

High spatial resolution techniques bear the potential to exclude surface contamination from coccolith analyses. For example, analysis by a conventional SIMS is conducted in repeated cycles, effectively revealing the depth profile of elements within an individual coccolith. Thus, the influence of surface contamination can be identified if the measured signal exceeds a certain threshold or if the depth profile does not stabilize during the analysis. This approach was successfully used to determine accurate Mg/Ca and Sr/Ca ratios in coccoliths^42^. Imaging methods such as NanoSIMS or TOF–SIMS provide an additional advantage as their spatial resolution may be sufficient to laterally resolve contaminating particles that are smaller than the coccolith. This creates the possibility to remove the influence of particles by data processing even if they were not removed from the sample before analysis.

Here we used NanoSIMS to quantify trace element content in individual coccoliths of *E.* *huxleyi* collected from the environment and from culturing experiments. Our focus was on Na/Ca and K/Ca, but we also determined individual coccolith Mg/Ca, Sr/Ca and Ba/Ca ratios in order to allow comparison with previous studies as well as explore potential differences between biologically essential (e.g., Mg and K) and non-essential (e.g., Sr) elements. Our aims were to (i) assess the effectiveness of data processing in removing the influence of surface contamination, (ii) investigate compositional variability within and between individual coccoliths, and (iii) explore whether there is a relationship between the Na and K content in the coccolith calcite and the parameters characterizing the environment where the coccoliths formed. To this end, we measured samples collected along an East–West transect of the Mediterranean Sea and from one location in the Black Sea, and samples obtained from *E.* *huxleyi* cultures grown at controlled salinities and alkalinities.

## Results

### NanoSIMS imaging of individual coccoliths

**Fig. 1:**
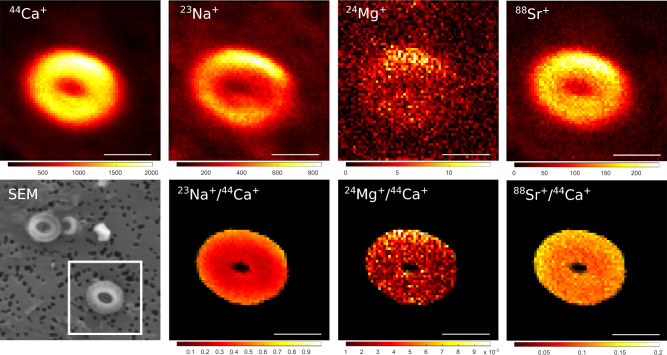
Example nanoSIMS images of an *E. huxleyi* coccolith placed on a polycarbonate filter. The specimen was collected from the Mediterranean Sea, station EM-6. Shown are images of ion counts accumulated over 1000 planes (upper panels), the corresponding images of ion count ratios (lower panels), and the SEM image. The scale bar is 2 μm. Note that the visibility of the noise on the filter due to low Ca counts was suppressed by setting the colour to black in pixels where the Ca ion counts fell below a certain threshold. Secondary ions ^39^K^+^ and ^138^Ba^+^ were not measured for this particular sample.

**Fig. 2:**
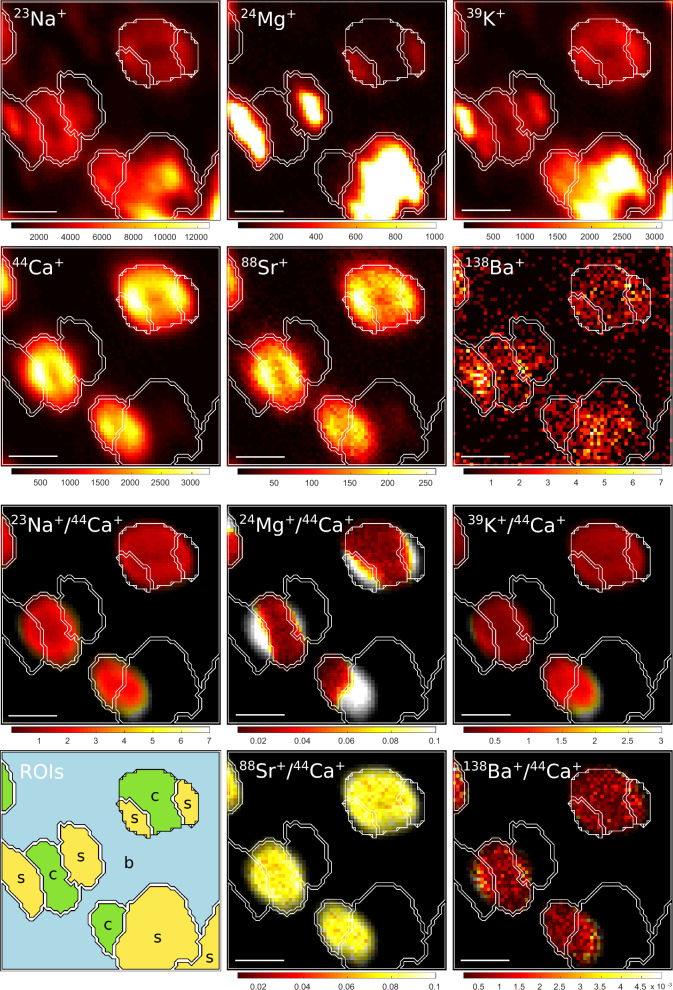
Example nanoSIMS images of *E. huxleyi* coccoliths placed on a polycarbonate filter. The specimens were collected from culture C6. Shown are images of ion counts accumulated over 1000 planes (panels in rows 1–2), the corresponding images of ion count ratios (panels in rows 3–4), as well as an image with regions of interest (ROIs) identified as coccoliths (c), contamination (s), and filter background (b). ROIs identified as contamination were drawn based on their conspicuous enrichment in Na, Mg, K and Ba relative to Ca. The scale bar is 2 μm. Note that the visibility of the noise on the filter due to low Ca counts was suppressed by setting the colour to black in pixels where the Ca ion counts fell below a certain threshold.

Coccoliths of *E.* *huxleyi* were easily identified in the NanoSIMS images as regions with significantly higher counts of ^44^Ca^+^, ^23^Na^+^, ^24^Mg^+^, ^39^K^+^ and ^88^Sr^+^ compared to the counts on the filter (Fig. 1 and 2). Given the size of the primary ion beam (350–500 nm) compared to that of the coccoliths (3–5 µm), the images revealed no conspicuous lateral variability within the coccolith. In some images, however, we observed regions adjacent to or overlapping with coccoliths, where the counts of ^24^ Mg^+^, and often also of ^23^Na^+^, ^39^K^+^, and ^138^Ba^+^, but not of ^88^Sr^+^, were elevated relative to the counts of ^44^Ca^+^ (Fig. 2). The presence of these regions indicates that contamination, probably related to salt precipitates or organics, played a role during the measurement and needed to be dealt with to minimize its influence on the accuracy of the measured trace element content in individual coccoliths.

In addition to the variability in the lateral direction, the accuracy of the element-to-calcium ratio (El/Ca) determination in an individual coccolith was also influenced by the variation of the corresponding El/Ca ion count ratio with depth. This variation could be due to surface-bound contamination, but also due to an incomplete stabilization of the secondary ion yields during the pre-sputtering phase of the measurement, which could be examined because the measurement of each individual coccolith involved repeated measurements over 1000 cycles (planes). Detailed analysis revealed that the variability with depth, quantified as a coefficient of variation (CV), was quite substantial, varied considerably among individual coccoliths, and depended on the trace element. For example, it reached up to 60% for Sr/Ca, up to 80% for Mg/Ca, up to 105% for Na/Ca, up to 135% for K/Ca, and up to 370% for the Ba/Ca ion count ratios^58^.

**Fig. 3:**
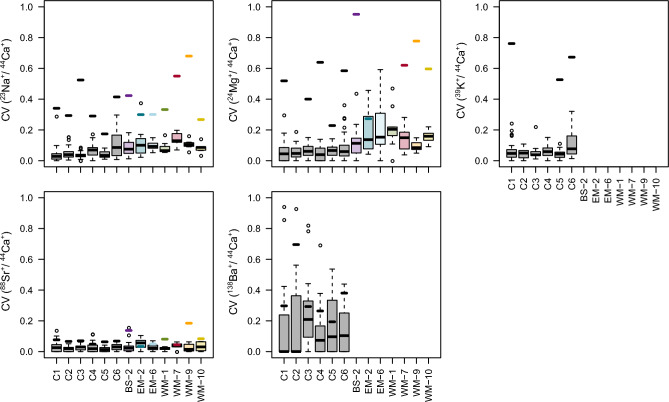
Visualization of the variability in the El/Ca ion count ratios within individual coccoliths and among coccoliths. The variability is expressed by the coefficient of variation (CV). The boxplots display the range of depth-related CV’s within coccoliths, thus the range of the within-lith variability. The thick horizontal bar indicates the CV among coccoliths from one sample, thus the within-sample variability of the coccolith-specific El/Ca ion count ratio. For samples where no horizontal bar is displayed, the within-sample CV exceeds 1. For each station (x-axis), the number of measured coccoliths is provided in Table 1.

To minimize the influence of the lateral and depth variability on the accuracy of the El/Ca ion count ratios measured in individual coccoliths, the pixels and planes in the NanoSIMS image stacks identified with contamination were excluded from the analysis. Although this exclusion led to a significant decrease in the variability of the El/Ca ion count ratios with depth, this variability could not be completely removed^58^. This residual variability with depth averaged at 7%, 9%, 6%, 3%, and 16% for the Na/Ca, Mg/Ca, K/Ca, Sr/Ca, and Ba/Ca ion count ratios, respectively (boxplots in Fig. 3). For Na/Ca and Mg/Ca, the residual variability was typically smaller in the cultured samples compared to the environmental ones, while it was similar and low for Sr/Ca in both types of samples.

The residual variability in the El/Ca ion count ratios with depth has two contributions, the “true” variability with depth and the variability due to the random nature of the secondary ion detection by NanoSIMS (the Poisson error). In our measurements, the Poisson errors were < 1% for the Na/Ca, Mg/Ca and K/Ca ion count ratios, < 4% for Sr/Ca, and reached up to 27% for Ba/Ca due to the generally very low amount of Ba ions detected from the coccoliths (Fig. 3). Thus, the Poisson errors had a negligible contribution to the precision of the El/Ca ion count ratio measurements for Na, Mg and K, while they contributed more for Sr, and dominated the measurement precision of the Ba/Ca ion count ratios. Barium data were excluded from further analysis because of the high Poisson error.

### Variability among coccoliths in a sample

El/Ca ion count ratios were highly variable among individual coccoliths within a sample. Smallest inter-coccolith variability was observed for Sr/Ca (< 10% for most samples), while the variability in Na/Ca and Mg/Ca ranged between 20 − 70% for most samples and even exceeded 80% for some samples (thick horizontal bars in Fig. 3). Inter-coccolith variability in K/Ca, which was only determined in cultured coccoliths, was large (> 50%). For Sr, the inter-coccolith variability was similar to the residual variability, suggesting tightly controlled incorporation, while inter-coccolith variability for Na, K and Mg was larger than the residual variability (Fig. 3), indicating heterogeneous incorporation.

### Coccolith El/Ca ratios and environmental conditions

**Fig. 4:**
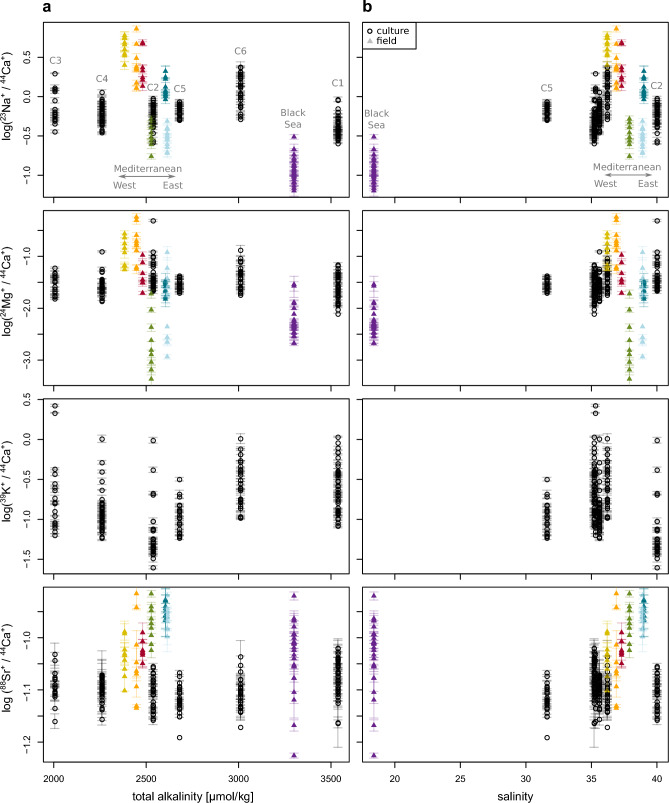
Log-transformed El/Ca ion count ratios in individual *E. huxleyi* coccoliths plotted against (**a**) total alkalinity and (**b**) salinity of the environments where they formed. Coccoliths originated from the Mediterranean and Black Sea (coloured triangles) and from laboratory cultures (black circles). Colour coding of the field samples corresponds to the colour coding of the sampling stations shown in Fig. 6, while codes C1–C6 correspond to the culture conditions shown in Figure S-1. Data points and error-bars indicate, respectively, the best estimate and precision (uncertainty) of the El/Ca ion count ratio in individual coccoliths.

**Fig. 5:**
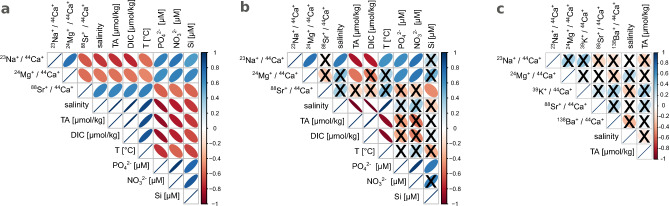
Correlation matrices between the log-transformed El/Ca ion count ratios in coccoliths and the relevant environmental parameters. Correlation coefficients were calculated using (**a**) only the data for the Mediterranean Sea coccoliths, (**b**) data for all environmental coccolith samples, i.e., from both the Mediterranean and Black Sea, and (**c**) data for the cultured coccoliths. Significant (*p* < 0*.*05) versus non-significant relationships are indicated by the absence and presence of a bold X, while the ellipse and colour visualize the correlation coefficient (*r*).

The log-transformed El/Ca ion count ratios were significantly different among the different samples of *E.* *huxleyi* coccoliths (Table S-1), and a Tukey post-hoc test identified which pairs of samples were significantly different from each other (see letters in parentheses in Table 1). However, analyses of the log-transformed El/Ca ion count ratios in the coccoliths versus environmental parameters did not reveal any clear relationships. On the one hand, the log-transformed Na/Ca and Mg/Ca ion count ratios in the Mediterranean coccoliths were significantly negatively correlated with alkalinity and salinity, while the corresponding correlations were significantly positive for Sr/Ca (Fig. 4 and 5a). On the other hand, when data for all environmental coccoliths were tested (i.e., including Black Sea samples as well), the correlation with alkalinity remained significantly negative for Na/Ca and Mg/Ca, but the correlations with salinity, although remaining significant, changed from negative to positive (for Na/Ca), or became insignificant (*p* > 0*.*05; for Mg/Ca and Sr/Ca; Fig. 5b). Because some of the environmental parameters were, to a variable degree, inter-correlated, significant correlations were detected also between the log-transformed Na/Ca, Mg/Ca and Sr/Ca ion count ratios and environmental parameters such as temperature or nutrient concentrations (Fig. 5a).

**Table 1:** Trace element content of coccoliths of *E. huxleyi.* Values are given as averages of N individual coccoliths per sample ± 1 SE.

nanoSIMS ion count ratios																					
Sample ID	**N**	^**23**^**Na/**^**44**^**Ca**^**†**^				^**24**^**Mg/**^**44**^**Ca**^**†**^				^**39**^**K/**^**44**^**Ca**^**†**^				^**88**^**Sr/**^**44**^**Ca × 1000**^**†**^				^**138**^**Ba/**^**44**^**Ca × 1000**^**†**^			
C1	43	0.437	±	0.003	(c)	0.0295	±	0.0004	(c)	0.307	±	0.005	(a)	84.2	±	0.2	(a)	1.68	±	0.01	(b)
C2	32	0.61	±	0.006	(b)	0.059	±	0.003	(a)	0.102	±	0.006	(c)	78.1	±	0.2	(b)	0.423	±	0.009	(e)
C3	18	0.8	±	0.02	(b)	0.0321	±	0.0007	(abc)	0.42	±	0.04	(ab)	81.1	±	0.3	(ab)	0.96	±	0.02	(c)
C4	36	0.629	±	0.005	(b)	0.0298	±	0.0005	(c)	0.164	±	0.005	(b)	79.8	±	0.1	(b)	2.86	±	0.02	(a)
C5	22	0.676	±	0.005	(b)	0.0295	±	0.0003	(bc)	0.117	±	0.003	(bc)	76.4	±	0.2	(b)	0.594	±	0.005	(d)
C6	27	1.34	±	0.02	(a)	0.045	±	0.001	(ab)	0.344	±	0.009	(a)	78.4	±	0.2	(b)	0.73	±	0.01	(d)
BS-2	28	0.129	±	0.002	(q)	0.0068	±	0.0002	(qr)	n.d				93.6	±	0.5	(n)	n.d			
EM-2	9	1.35	±	0.04	(o)	0.0252	±	0.0008	(op)	n.d				112.6	±	0.4	(m)	n.d			
EM-6	11	0.306	±	0.008	(p)	0.026	±	0.003	(pq)	n.d				109.4	±	0.5	(m)	n.d			
WM-1	9	0.34	±	0.01	(p)	0.0044	±	0.0007	(r)	n.d				107	±	1	(m)	n.d			
WM-7	7	2.7	±	0.2	(n)	0.049	±	0.004	(no)	n.d				94.9	±	0.6	(mn)	n.d			
WM-9	9	2.9	±	0.2	(n)	0.24	±	0.02	(m)	n.d				91	±	2	(n)	n.d			
WM-10	9	4.4	±	0.1	(m)	0.132	±	0.009	(mn)	n.d				91.8	±	0.9	(n)	n.d			

**molar ratios [mmol/mol]**^‡^ – **calibrated using calibration relation of synthetic calcites**^58^																					
Sample ID	**N**	**Na/Ca**				**Mg/Ca**				**K/Ca**				**Sr/Ca**				**Ba/Ca × 1000**			
C1	43	11.14	±	0.09		4.29	±	0.05		n.d				2.658	±	0.005		24.5	±	0.2	
C2	32	15.6	±	0.1		8.6	±	0.4		n.d				2.464	±	0.005		6.2	±	0.1	
C3	18	20.5	±	0.6		4.7	±	0.1		n.d				2.56	±	0.01		14	±	0.2	
C4	36	16	±	0.1		4.34	±	0.008		n.d				2.52	±	0.003		41.6	±	0.3	
C5	22	17.2	±	0.1		4.3	±	0.04		n.d				2.411	±	0.007		8.65	±	0.08	
C6	27	34.2	±	0.5		6.5	±	0.1		n.d				2.474	±	0.006		10.7	±	0.2	
BS-2	28	3.28	±	0.05		0.99	±	0.03		n.d				2.95	±	0.02		n.d			
EM-2	9	35	±	1		3.7	±	0.1		n.d				3.55	±	0.01		n.d			
EM-6	11	7.8	±	0.2		3.8	±	0.5		n.d				3.45	±	0.02		n.d			
WM-1	9	8.6	±	0.3		0.6	±	0.1		n.d				3.37	±	0.03		n.d			
WM-7	7	69	±	5		7.2	±	0.6		n.d				3	±	0.02		n.d			
WM-9	9	74	±	6		34	±	3		n.d				2.88	±	0.06		n.d			
WM-10	9	113	±	3		19	±	1		n.d				2.9	±	0.03		n.d			

^**†**^Letters in parentheses indicate groups of significant mean sample difference (*p* < 0.05) determined using Tukey’s honest significance test. Cultured and environmental samples were compared separately (groups a − e for cultures, groups m − r for environmental samples). n.d. = not determined.

^‡^Note that the overall accuracy of the molar ratios is governed by the calibration relation, which is at best ± 20%, ± 21%, ± 5%, and ± 4% for Na/Ca, Mg/Ca, Sr/Ca, and Ba/Ca, respectively.

El/Ca ion count ratios measured in the cultured coccoliths varied in a similar range as those from the environmental samples (Fig. 4). ANOVA revealed significant differences among cultures (Supplementary Table S-1), and the Tukey post-hoc test identified which cultures were significantly different from the other cultures (see letters in parentheses in Table 1). However, none of the log-transformed El/Ca ion count ratios significantly correlated with the total alkalinity or salinity of the culture media (Fig. 5c). When comparing average El/Ca ion count ratios per culture (Table 1), Sr/Ca showed the lowest variability among cultures (CV of about 3%), while the variability among cultures was at least tenfold greater for the other trace elements (CV ranging from 32% for Mg/Ca to 55% for K/Ca).

### Molar El/Ca ratios in coccolith samples

El/Ca ion count ratios in the studied *E. huxleyi* coccoliths were converted to molar El/Ca ratios using the calibration relations obtained for two synthetic calcites^58^. Considering data for all measured coccoliths, molar El/Ca ratios varied between 1*.*9–3*.*8 mmol mol^−1^ for Sr, 0*.*003–0*.*077 mmol mol^−1^ for Ba, 1*.*6–186 mmol mol^−1^ for Na, and 0*.*06–83 mmol mol^−1^ for Mg. Molar ratios averaged over coccoliths from a specific site or culture are listed in Table 1. Due to the uncertainty of the calibration relations, the accuracy of the reported values is not better than ± 5% for Sr/Ca, ± 4% for Ba/Ca, ± 20% for Na/Ca, and ± 21% for Mg/Ca. The low measurement precision for Ba/Ca (due to low Ba counts; Fig. 2) eventually limited the accuracy of single coccolith Ba measurements. The measured K/Ca ion count ratios could not be converted to molar ratios due to an unknown K content of the synthetic calcites used for the calibration.

## Discussion

In this study we explored the use of NanoSIMS as a tool to quantify trace element content in individual coccoliths of *E. huxleyi*. The overall approach involved deposition of the coccoliths on a polycarbonate filter substrate, cleaning by extensive rinsing with a pH buffer, drying, the actual NanoSIMS measurement, extensive data processing to minimize the influence of contamination on the accuracy of the measured El/Ca ion count ratios, and finally the conversion of the ion count ratios to molar ratios using calibration relations obtained for custom-made synthetic calcites. Our main aims were to quantify the variability within and between individual coccoliths and to assess whether the Na and K content of *E. huxleyi* coccoliths, which were measured in this study for the first time, could be linked to parameters characterizing the environment where the coccoliths formed. Trace element incorporation into biominerals is governed by inorganic chemical processes (e.g., the compatibility of the trace element with a mineral phase) as well as various biological process, i.e., vital effects such as the open vs. closed nature of the site of calcification^59,60^. Trace element systematics of biominerals may thus shed light on calcification mechanisms^44,59^. Calcification in coccolithophores is under tight biological control: trace element concentrations are low and differ from those in inorganically produced calcites^59^, and calcification occurs intracellularly in a tightly controlled environment maintained by selective membrane transport.

### Variability within and between individual coccoliths

Using a beam size of 350–500 nm, which is about 10% of the coccolith size, our NanoSIMS measurements revealed no conspicuous spatial variability in the trace element content within individual coccoliths (Figs. 1 and 2). The areas of elevated counts of Mg, Na, and K, which we often observed overlapping with or next to coccoliths, were interpreted as contamination. After removal of these surface contaminants via data processing, the spatial distributions of Sr, Mg, Na and K in *E. huxleyi* coccoliths were homogenous within the resolution of our measurements. This homogenous spatial distribution would be consistent with intracellular element incorporation into coccoliths^61^. Previous studies utilizing synchrotron techniques with a higher spatial resolution (10 − 100 nm) revealed, however, both homogenous and heterogeneous spatial distributions within coccoliths from cultures and fossil coccolithophores^43,45^, including stripe-like Sr concentration patterns in the lopadoliths of *Scyphosphaera apsteinii*^62^. Accordingly, further studies are required to assess whether coccoliths are internally homogenous or exhibit heterogeneity, and this may depend on the element, species, and nature of the sample (fossil, extant or cultured).

In contrast to undetectable lateral variability, we did observe a residual variability in El/Ca ion count ratios with depth in the coccoliths, averaging between 3 − 16% depending on the trace element. However, this variability likely reflected the progressive change in the ionization efficiency of the measured secondary ions during the measurement, which is known to depend on the element and on the concentration of primary ions implanted into the analysed material. This variability could not be avoided because of the very small thickness of the coccoliths; the limited amount of available material did not allow for longer pre-sputtering of the coccoliths, which could have improved the stability of the secondary ion yields. Thus, we considered this residual depth variability as the precision of the measured El/Ca ion count ratios.

One of the most striking results of the present study is the high variability among individual coccoliths within a sample with respect to their trace element content. The lowest variability was observed for the Sr content, with inter-coccolith variability close to the precision of the measurements, consistent with XANES evidence that Sr resides in a Ca site in the calcite lattice^62,63^. However, the inter-coccolith variabilities for Na, K, and Mg were much higher, outside of the measurement precision (Fig. 3), suggesting that these elements may reside in organics, interstitial sites or are incorporated differently. Moreover, Sr is a non-essential biological element while Mg and K are essential for many cell functions. Previous studies showed that *E. huxleyi* contains a Ca-P concentrated compartment^46,63,64^. This intracellular pool transfers Ca and Sr to the coccolith vesicle where calcification occurs, followed by the extrusion of the formed coccolith to the cell surface^63^. Our observations of low variability in Sr/Ca and higher variability in Na/Ca, K/Ca and Mg/Ca are consistent with a calcification mechanism where Sr and Ca are both transferred from the intracellular pool to the calcification vesicle, whereas other trace elements (Na, K and Mg) are incorporated only after coccolith extrusion.

### Variability in trace element content with environmental parameters

Variability in El/Ca ratios among coccoliths from different environments and cultures was distinctly higher than among coccoliths from the same sample because environmental factors contribute as well. Sr/Ca ratios among the measured coccoliths varied by a factor of two, in line with the narrow range of Sr/Ca ratios found in other studies^16,19,65^. Variability for the other elements was much greater, with the lowest and highest trace element contents spanning two (Na and K) to three (Mg) orders of magnitude (Figs. 3 and 4). It appears that variability of a biologically non-essential element such as Sr is much lower than that of biologically essential elements such as Mg and K^29^.

Despite the large variability in trace element content among *E. huxleyi* coccoliths from the same sample, statistical analysis revealed significant differences among samples collected from different locations or cultured under different conditions. However, plots of the El/Ca ion count ratios in the coccoliths against environmental parameters did not reveal any clear relationships. When only the coccoliths collected from the Mediterranean Sea were considered, Na/Ca and Mg/Ca ion count ratios showed a decreasing trend with increasing salinity and alkalinity, while the Sr/Ca ion count ratios increased. However, most of these trends were not followed by the *E.* *huxleyi* coccoliths collected from the Black Sea, except for the trend of a decreasing Na and Mg content with increasing alkalinity.

It is important to emphasize, however, that parameters characterizing environmental samples are often inter-correlated. For instance, seawater salinity, total alkalinity and temperature increase along the West–East transect of the Mediterranean Sea, while nutrient concentrations decrease. Thus, the apparent relationships between the Na/Ca, Mg/Ca and Sr/Ca ion count ratios in the Mediterranean *E. huxleyi* coccoliths and the corresponding seawater salinity or total alkalinity may be a reflection of a relationship with another environmental parameter, e.g., the concentration of nutrients or a parameter that has not been measured in this study.

This hypothesis is supported by the El/Ca ion count ratios measured in the coccoliths of the cultured *E.* *huxleyi*. On the one hand, the El/Ca ion count ratios measured in the cultured coccoliths varied in a similar range as those from the environmental samples, and the coccoliths from some of the cultures did show significant differences in their El/Ca ratios in comparison to the other cultures. Although the salinities and total alkalinities of the culture media varied in a similar, or even wider, range than their environmental counterparts, the El/Ca ion count ratios showed no specific trends with total alkalinity or salinity for any of the measured trace elements. Multiple factors impact incorporation of trace elements^59,60^, and these additional factors may mask any effects of salinity or alkalinity. For example, Sr/Ca in coccoliths depends on the growth and calcification rate^16^, and because the growth rates in our cultures were rather uniform (Table S-3, Figure S-3), this could be a reason why we only observed minimal differences in average Sr/Ca values across the different cultures.

Taken together, the present data suggest that the incorporation of trace elements Na, Mg, K and Sr into coccoliths of *E.* *huxleyi* is, to a large degree, biologically controlled, and that the variability between coccoliths originating from different environments is directly or indirectly driven by an environmental parameter that was not identified in this study.

### Trace element content in individual coccoliths

Ion count ratios are quantitative but do not bear information about actual element concentrations because of element- and matrix-dependent ionization efficiencies and instrumental fractionation. To enable the comparison of our data with previous studies, the measured El/Ca ion count ratios were therefore converted to molar El/Ca ratios using two custom-made synthetic calcites with known bulk concentrations of Na, Mg, Sr and Ba (CalUUa and CalUUb^58^). Unfortunately, K contents of these standards are not available. Because of the uncertainty in the calibration parameters, the accuracy of the reported trace element concentrations is not better than ± 5% for Sr, ± 4% for Ba, ± 20% for Na, and ± 21% for Mg. Additionally, the overall uncertainty of the molar ratios is likely even higher because the synthetic inorganic calcites were not completely matrix-matched with the biogenic calcites formed by coccoliths. Matrix-matching between standards and samples is, however, extremely difficult, if possible at all for biogenic calcites.

Strontium content in individual coccoliths ranged between 1*.*9–3*.*8 mmol mol^−1^ (median of 2*.*8 mmol mol^−1^), which is consistent with the narrow range of 2*.*0–3*.*7 mmol mol^−1^ reported in the literature^16,19,65^. Average magnesium contents ranged between 0*.*6–34 mmol mol^−1^ (median 4*.*3 mmol mol^−1^; with a range for individual coccoliths of 0*.*06–83 mmol mol^−1^). Again, this is in line with the large variability and range of previously reported Mg content in coccoliths (from 0*.*01 mmol mol^−1^ up to 98 mmol mol^−1^^ 19,20,27,56,66–68^). The lowest Mg contents were determined in earlier studies using extensive cleaning protocols^19,20,57^. Mg contamination from organic matter and clays was noted as a serious problem for accurate Mg determination in coccolith calcite^42,55^. The highest reported coccolith Mg/Ca ratios were obtained from experiments with elevated CO_2_ concentrations^68^. High variability in Mg/Ca was also observed between different strains of *E.* *huxleyi*^66^. Average barium contents ranged between 0*.*006–0*.*04 mmol mol^−1^ (median 0*.*01 mmol mol^−1^), which is slightly lower than the range of 0*.*02–0*.*12 mmol mol^−1^ determined by Langer et al.^22^ However, the coccoliths measured by Langer et al.^22^ were grown in a culture medium that was more than 10 times enriched in Ba with respect to seawater Ba concentrations.

The only data on potassium content in coccoliths reported to date were measured by synchrotron X-ray fluorescence spectrometry in overgrowth-free and overgrown regions of two single fossil coccoliths of *Watznaueria Britannica*^45^. The reported values for K/Ca ranged between 0*.*1–24*.*9 mmol mol^−1^, and higher values were associated with overgrown regions. In the present study, ion count ratios of K/Ca determined on individual *E.* *huxleyi* coccoliths ranged over 2 orders of magnitude, consistent with the reported range of values, but they could not be converted into molar ratios in the absence of a proper calibration.

Sodium content in the studied coccoliths was highly variable, with averages ranging between 3–113 mmol mol^−1^ (median 17 mmol mol^−1^, with a range for individual coccoliths of 1*.*6–186 mmol mol^−1^). To the best of our knowledge, these are the first reported data on Na content in coccoliths. Compared to the other trace elements in coccoliths and to Na content in other calcifying organisms such as foraminifera^48–51^, these molar Na/Ca ratios are rather high. This difference between small coccoliths and larger foraminifera can be due to (i) sea salt contamination, (ii) surface topography, or (iii) differences in biomineralization pathways. Our extensive sample cleaning procedure combined with data processing might have failed to completely remove contamination with sea salt. However, Na/Ca ratios determined in coccoliths from the Mediterranean Sea were anti-correlated with seawater salinity. Another indication for the role of contamination with sea salt in our study is the lack of detectable Na or K in coccoliths using STEM-EDX spectroscopy^46^. However, as these authors did not detect Mg in the coccolith calcite either, it is likely that Na and K were below the detection limit of the STEM-EDX method.

A second possible explanation for the rather high Na content in coccoliths is uneven surface topography, which is known to impact secondary ion yields in SIMS measurements^69,70^. The thickness of a coccolith, i.e., the distance between the proximal and distal shield elements, is about 500 nm^71^, which is comparable to the amplitude of surface roughness in polished shells of foraminifera studied by Geerken et al.^72^ The study by Geerken et al.^72^, however, showed that the major variability in the elemental distribution over foraminiferal shell walls did not correlate with topography of this amplitude. Furthermore, based on studies that used NanoSIMS to measure trace element distributions in forams embedded in a resin (e.g., Ref.^72–74^), we would expect to measure elevated El/Ca ion count ratios at the edges along the inner and outer perimeter of the coccoliths, which is where the abrupt transition in height between the coccolith and the underlying substrate (filter) occurs, and this was not observed.

Differences in calcification between foraminifera and coccolithophores could be a third explanation, because calcification mechanisms differ in many ways^59,60^, with coccolith calcite being low in most trace elements compared to foraminifera because of highly controlled intracellular calcification. The relatively high Na/Ca ratio of coccoliths compared to foraminifera and other calcifiers requires further study.

## Conclusions

In this study, we used NanoSIMS to determine, for the first time, Na/Ca and K/Ca ratios in individual coccoliths of *Emiliania huxleyi*. We developed a data processing workflow that minimized the influence of contamination on the measurement accuracy and precision. Along with the Na/Ca and K/Ca ratios, we also determined Mg/Ca and Sr/Ca ratios in the same coccoliths to be able to compare our results with published data. The Mg/Ca and Sr/Ca ratios of individual *E.* *huxleyi* coccoliths ranged between 0*.*06–83 mmol mol^−1^ and 1*.*9–3*.*8 mmol mol^−1^, respectively, which are in line with Mg/Ca and Sr/Ca determined in previous studies. Both Na and K appear to be homogeneously distributed within the coccolith calcite and highly variable among individual coccoliths. Na/Ca ratio ranged between 1*.*6–186 mmol mol^−1^ among individual coccoliths, whereas the measured ^39^K^+^/^44^Ca^+^ ion count ratios (which could not be converted to molar K/Ca ratios due to unavailable calibration standard) varied between 0*.*03–2*.*7. We found significant differences in the Na and K content among *E.* *huxleyi* coccoliths with different origin (sampled from six sites in the Mediterranean Sea and one site in the Black Sea, or grown in laboratory cultures under different salinities and total alkalinities). However, these data did not allow us to identify an empirical or mechanistic relationship between the Na or K content in coccoliths and the parameters characterizing the environment where they formed. Before coccolith Na/Ca and K/Ca ratios can be used in proxy studies of past environments, we have to further advance our understanding of the biological processes governing the incorporation of these and other trace elements in coccolithophores.

## Materials and methods

### Field sampling

**Fig. 6:**
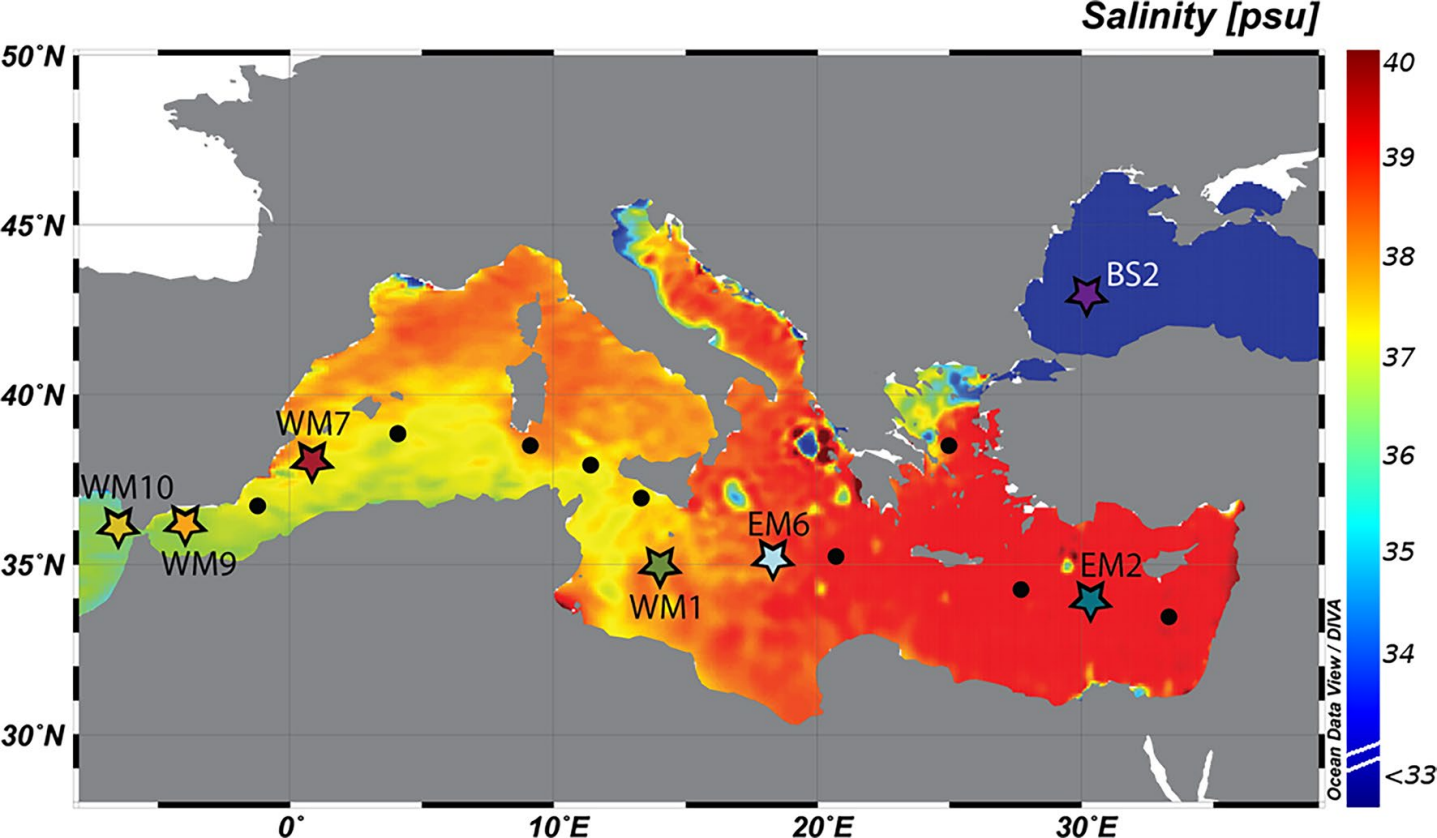
Sampling stations during cruises 64PE401, 64PE406 and 64PE407 of the R/V Pelagia. Black dots and filled stars indicate sampled and analysed stations, respectively. Locations are superimposed on the salinity map of the surface waters in the Mediterranean and Black Sea. The map was created with Ocean Data View 4.6.5^84^ by extrapolation (DIVA-gridding) of the MedAtlasII dataset^85^.

Samples of *Emiliania huxleyi* coccoliths were collected during cruises 64PE401, 64PE406, and 64PE407 of the R/V Pelagia in September 2015, January 2016, and February 2016, respectively. At all stations (Fig. 6), seawater was retrieved from surface waters (water depth of 25–30 m) using a CTD rosette sampler. On cruise 64PE401 (Black Sea), volumes of approximately 1 L were filtered through in-line polycarbonate filters (Millipore; pore size 0*.*4 µm and diameter 47 mm) that were pre-coated with a 20 nm thick Au layer (see below). The filters were subsequently stored frozen (− 20 °C) onboard the ship. Upon arrival at the laboratory in Utrecht, NL, the filters were transferred in a frozen state to a filtration setup and rinsed three times with a 0*.*05 M NH_3_HCO_3_ buffer (pH 7.8). Subsequently the filters were dried in an oven at 45 °C for 24 h and stored in a desiccator until analysis by NanoSIMS. Sampling on cruises 64PE406 and 64PE407 (Mediterranean Sea) involved similar steps, except the seawater volumes (0*.*1–1 L) were first pre-filtered through 5 µm pore-sized polycarbonate filters to remove larger plankton, and filters were not pre-coated. The samples were rinsed with a 0*.*05 M NH_3_HCO_3_ buffer (pH 7.8) before being stored frozen (− 20 °C) during transport, after which they were dried and stored likewise the others.

In addition to coccolith sampling, the retrieved seawater was sampled for the analyses of dissolved inorganic carbon (DIC), total alkalinity (TA), and nutrients (Si, P and N species). DIC and TA were measured onboard the ship during cruises 64PE406 and 64PE407 using the Versatile Instrument for the Determination of Titration Alkalinity (VINDTA) model 3C (Marianda GmbH, Kiel, Germany). During cruise 64PE401, pH was measured, and TA was determined by titration onboard the ship. All water samples for nutrient analyses were sterile-filtered. For the determination of N and P, the samples were stored frozen (− 20 °C), while for Si they were stored cooled (4 °C) and in the dark until analysis.

### Culturing of E. huxleyi

The culture media were designed in such a way that alkalinity and salinity, which covary in natural seawater, were decoupled from each other and kept constant during the growth of *E. huxleyi* cells. Six culture conditions were chosen at three salinities and four alkalinities, such that alkalinities varied at the intermediate salinity and salinities varied at the lower-intermediate alkalinity (Figure S-1). Other parameters that may affect element incorporation, such as temperature and light conditions, were kept constant and the same for all cultures.

Culture media were prepared by diluting aged oligotrophic North Sea water with ultrapure water to an initial salinity of approximately 30, followed by the addition of NaCl, KHCO_3_ and K_2_CO_3_ to achieve different salinities and alkalinities. After addition of NaCl, the media were autoclaved, and the evaporated water was replaced by autoclaved ultrapure water. TA was determined in each medium, and the amounts of KHCO_3_ and K_2_CO_3_ required to reach the target alkalinities were calculated using the R package *seacarb*^75^. KHCO_3_ and K_2_CO_3_ were added after autoclaving, since autoclaving can seriously alter the carbonate chemistry^76^. Finally, the media were purged with sterile air for 24 h to equilibrate with ambient conditions. Nutrients were added such that P and N were at K/10, while trace metal and vitamin solutions were at K/2 concentrations in the final media (following Ref.^77^). The media were sampled for pH, salinity and TA determination at the beginning and end of the experiments to verify that the conditions remained approximately constant. Salinity was measured using a VWR CO310 handheld conductivity meter calibrated to seawater salinity. TA was determined photometrically on 60 ml samples using an automated spectrophotometric alkalinity system (ASAS) as previously described^78^.

Batch cultures were prepared using the calcifying *E. huxleyi* strain RCC2050 isolated from the Mediterranean Sea (34°8’N, 18°27’E) in 2008. Before culturing, the stock culture was kept at 15 °C with a 16 h:8 h light:dark cycle at a light intensity of 10–20 µmol photons m^-2^ s^-1^ in the culture collection at NIOZ Texel, NL. Each batch culture was inoculated such that the starting cell density was about 40 cells mL^−1^. A total of 5 transfers into a fresh medium were carried out to ensure adaptation of the strain to the culture medium and to minimize the carry-over of coccoliths. The transfers were performed when the cell density approached 50,000 cells mL^−1^ to ensure stable carbonate system conditions^79^. During the experiment, the salinity and the carbonate system parameters (TA, pH and *p*CO_2_) of the media were reasonably stable (Figure S-2 and Table S-2). Furthermore, the cultures were monitored daily for cell vitality. Cell density was determined with flow cytometry, and cells were inspected by light microscopy for coccolith production. Specific growth rates for each batch culture were calculated by linear regression from the log-transformed cell densities (*R*^2^ of the regressions were generally > 0*.*99; Table S-3 and Figure S-3) and ranged between 0*.*42–0*.*5 d^−1^.

The cultures were harvested during the exponential growth phase at densities of 35,000–40,000 cells mL^−1^. Coccoliths were collected by filtration of a small aliquot (1–3 mL) through Au-coated polycarbonate filters (0*.*4 µm pore-size, 27 mm diameter) and rinsed extensively with approximately 50 mL of a 0*.*05 M NH_3_HCO_3_ buffer (pH 7.8). The filters were dried in an oven at 45 °C for 3 h and stored in a desiccator until NanoSIMS analysis.

### NanoSIMS analysis

When possible, the polycarbonate filters were coated with a 10 nm thick Au layer prior to filtration. For NanoSIMS analysis, circular pieces (either 5 or 10 mm in diameter) were cut out from the dried filters, and the pieces were coated with an additional 10 nm thick Au layer. The coatings were applied using a JEOL JFC-2300HR high resolution sputter coater aided by a JEOL FC-TM20 thickness controller. The coated circular filter pieces were subsequently mounted in a NanoSIMS sample holder and imaged with a high-resolution table-top SEM (JEOL JCM-6000PLUS Neo Scope) to identify coccoliths suitable for NanoSIMS analysis. The SEM images were then used to locate the identified coccoliths in the NanoSIMS instrument using the built-in CCD camera and real-time secondary ion images of pre-sputtered fields of view.

The NanoSIMS imaging analyses were carried out with a Cameca NanoSIMS 50L instrument at Utrecht University using a duoplasmatron oxygen source with an 8 kV primary O^–^ beam and electron multiplier detectors tuned for ^23^Na, ^24^Mg, ^39^K, ^44^Ca, ^88^Sr, and ^138^Ba. The samples were analysed during four analytical sessions: August, November and December 2016 (field samples), June 2018 (cultured samples), and January 2019 (selected field and cultured samples). The measurement protocol involved two major steps, which were carried out in the same way during all sessions. First, a larger field of view (FOV) was pre-sputtered with the primary ion beam to remove as much of surface contaminants as possible and to achieve a stable secondary ion yield. This was then followed by the acquisition of images over a smaller FOV (between 5 µm × 5 µm and 20 µm × 20 µm in size) that excluded the rim of the pre-sputtered area. During imaging the primary ion beam size was 350–500 nm and the dwelling time was 1000 µs pixel^−1^. To increase the secondary ion counts, images were acquired over 1000 cycles (planes). Detailed settings of the NanoSIMS 50L instrument during pre-sputtering and image acquisition are listed in Table S-4.

### NanoSIMS data processing

**Fig. 7:**
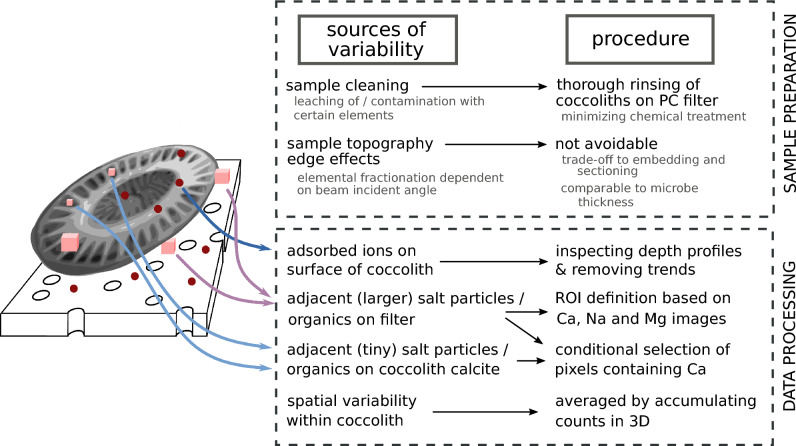
Scheme of the workflow employed during sample preparation and data processing. The aim was to obtain accurate and precise El/Ca ratio NanoSIMS measurements in coccolith calcite placed on polycarbonate filters.

NanoSIMS image stacks were processed with the Matlab-based freeware program Look@NanoSIMS^80^. A data processing workflow (Fig. 7) was developed to optimize the precision and accuracy of determination of the element-to-calcium (El/Ca) ion count ratios in individual coccoliths.

First, the images in the stack were aligned based on the ^44^Ca ion counts and accumulated. Using the accumulated images of ^44^Ca, ^23^Na and ^24^ Mg, regions of interest (ROIs) were drawn by hand and identified with coccoliths (regions rich in ^44^Ca) or with potential contaminants such as precipitates of sea salts or remnants of organics (regions rich in ^23^Na and ^24^ Mg). The remaining areas were identified as background and represented the polycarbonate filters.

In the second step, depth profiles of El/Ca ion count ratios were inspected in each coccolith ROI to assess whether insufficient pre-sputtering, surface-bound contamination, or complete ablation of the coccolith played an important role during the measurement and thus possibly affected the accuracy of the coccolith’s El/Ca determination. This was done by quantifying the variation of the El/Ca ion count ratios with depth by means of a coefficient of variance (CV) corrected for the variability due to the Poisson counting statistics (see Chapter 6 in Ref.^58^). When the depth variation was high, the depth profile was inspected in detail to identify the range of planes over which majority of the depth variation occurred. These planes were removed from the subsequent analysis. Thus, the El/Ca ratio per coccolith was determined as the ratio of ion counts accumulated over planes where the measured El/Ca ratios per plane showed minimal variation with depth.

In some coccolith ROIs, the minor elements were elevated in tiny distinct locations not spanning the whole depth domain that was analysed. We interpret these regions as particulate surface contamination, which can be located atop, underneath or in between the structural calcite elements of the coccolith (left panel in Fig. 7). During the measurement involving 1000 planes, such a particle would become sputtered away, which manifested itself by a gradual decrease in ion counts of ^23^Na, ^24^Mg or ^39^K, and a corresponding increase in ion counts of ^44^Ca, in pixels corresponding to the particle. If those pixels corresponding to the particle were removed from the analysis for all planes, the remaining number of pixels corresponding to the contamination-free coccolith would become less. This would lead to a decreased precision of the El/Ca ratio determination due to a lower number of ions detected per coccolith. To avoid this, the accumulation of ion counts of a given mass was adapted such that in each plane the counted ions were accumulated only in those pixels where ^44^Ca ions were detected. Because the ^44^Ca count rates were low, the probability of detecting zero ^44^Ca ions in any given pixel and plane was relatively high. To avoid biased results, the above condition was therefore applied not on single planes but on stacks of 5 accumulated consecutive planes^58^. Given the limitations imposed by the sensitivity of the NanoSIMS instrument and the small amount of material present in an individual coccolith, this approach maximized the number of ions detected from the coccolith while minimizing the influence of localized (particle-like) contamination, thus improving the overall precision and accuracy of the El/Ca determination in the coccolith.

In the last step, the coccolith El/Ca ion count ratios measured by NanoSIMS were converted to molar El/Ca ratios based on the calibrations determined for the synthetic CalUU calcites^58^. This was possible for the Na/Ca, Mg/Ca, Sr/Ca and Ba/Ca ratios, but not for the K/Ca ratio.

Using the instrument settings listed in Table S-4, all masses measured in this study could be well resolved except for ^88^Sr and ^138^Ba, which overlapped with the non-resolvable interference with the ^44^Ca_2_^+^ dimer, and the ^40^Ca_2_^26^Mg^16^O_2_^+^ multimer, respectively. The measured coccolith ^88^Sr/^44^Ca ratios were corrected for the contribution of the ^44^Ca_2_^+^ dimer, while the influence of the ^40^Ca_2_^26^Mg^16^O_2_^+^ multimer interference on the determination of the ^138^Ba/^44^Ca ratio was negligible^58^ and thus not considered during data processing.

### Statistical analyses

Statistical analyses were done in R^81–83^. Because the El/Ca ion count ratios determined by the NanoSIMS analysis were skewed towards higher values, they were log-transformed before performing the analyses. Analysis of variance (ANOVA) combined with post-hoc Tukey tests were performed to test for significant differences among coccolith samples. Correlation analyses were performed to assess relationships between the log-transformed El/Ca ion count ratios and environmental parameters.

## Supplementary Information

Supplementary Information.

## Data Availability

The datasets generated during and/or analysed during the current study are available from the corresponding author on reasonable request.
