# Supplementary material for: Sodium and potassium analysis of individual coccoliths by secondary ion mass spectrometry
Source: Sci Rep. 2026 Feb 28;16:11348. doi: 10.1038/s41598-026-40623-2 (PMC13048990; doi:10.1038/s41598-026-40623-2)
Supplement: Supplementary file 1 — Supplementary Information. [file 41598_2026_40623_MOESM1_ESM.pdf]

Supplementary Information

Sodium and potassium in individual coccoliths of *Gephyrocapsa huxleyi* (*Emiliania huxleyi*)

Anne Roepert, Jack J. Middelburg, Gabriella M. Weiss, Marcel T. J. van der Meer, Lubos Polerecky

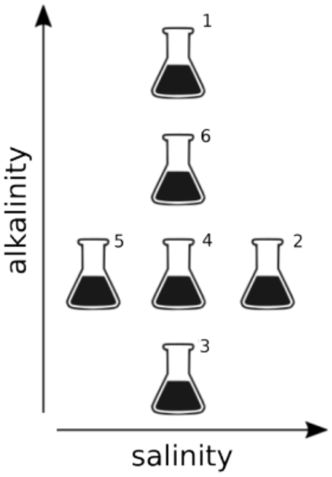

Figure S-1:
Scheme of the culturing media used to grow *E. huxleyi* cells. Salinity and alkalinity were uncoupled during the culture experiments. Salinities varied at a lower-intermediate alkalinity level, while alkalinities varied at an intermediate salinity level. The numbers indicate the culture condition C1−C6.

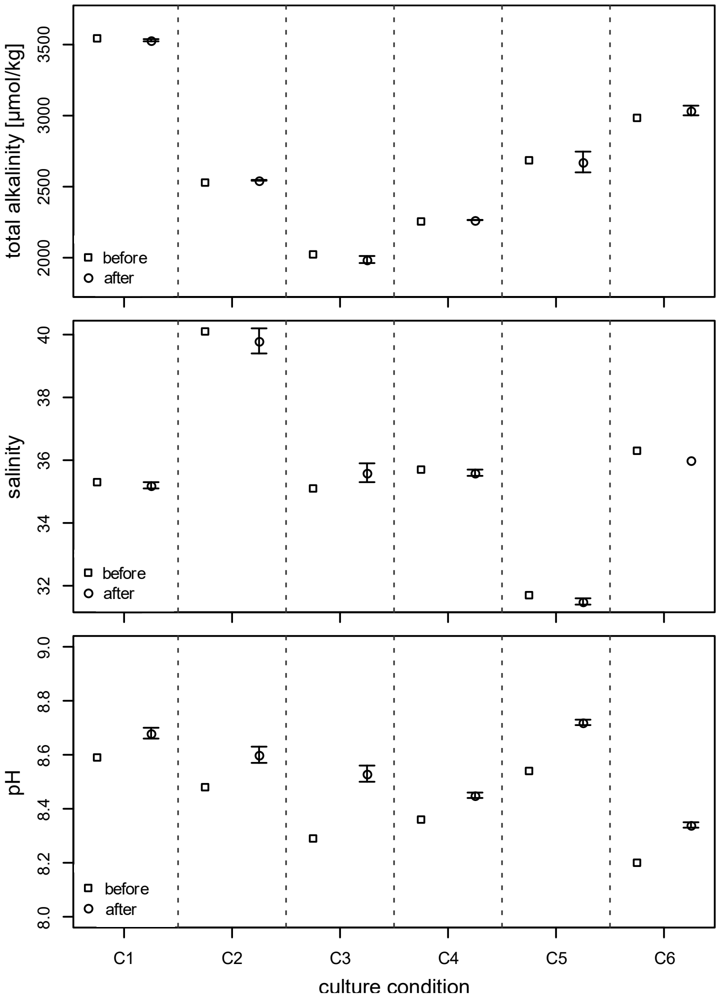

Figure S-2:

Chemical characteristics of the culturing media used to grow *E. huxleyi* cells. Shown are total alkalinity, salinity and pH of the media for the different experimental treatments before (squares) and after (circles) conducting the experiments with *E. huxleyi*. Error bars depict ±1SD of triplicate measurements.

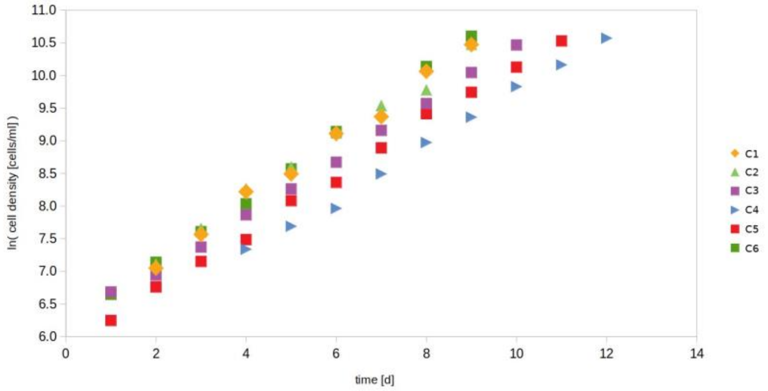

Figure S-3:

Growth of *E. huxleyi* cells. Shown are ln-transformed cell densities of the culture batches over time during the experiment. Specific growth rates per batch, μ in [d^{-1}], were obtained by regression and are given in Table S-3.

Table S-1:

Results of ANOVA testing for significant differences between samples from cultures and from the field.

	culture samples		field samples	
**ion count ratio**	*F* value	*p* value	*F* value	*p* value
$^{23}Na^+ / ^{44}Ca^+$	36.1	< 0.01	167.1	< 0.01
$^{24}Mg^+ / ^{44}Ca^+$	6.0	< 0.01	35.9	< 0.01
$^{39}K^+ / ^{44}Ca^+$	20.1	< 0.01	n.d.	n.d.
$^{88}Sr^+ / ^{44}Ca^+$	8.3	< 0.01	7.1	< 0.01
$^{138}Ba^+ / ^{44}Ca^+$	171.6	< 0.01	n.d.	n.d.

Table S-2:

Culture conditions of the media. Reported are average values ± 1 SD of measurements before and after conducting the experiments.

culture condition	pH	alkalinity [mol kg^{-1}]	salinity
C1	8.64 ± 0.06	3537 ± 9	35.3 ± 0.1
C2	8.54 ± 0.08	2537 ± 11	40.0 ± 0.2
C3	8.41 ± 0.17	2005 ± 25	35.4 ± 0.4
C4	8.41 ± 0.06	2260 ± 7	35.6 ± 0.1
C5	8.63 ± 0.13	2680 ± 8	31.6 ± 0.1
C6	8.27 ± 0.10	3010 ± 37	36.2 ± 0.2

Table S-3:

Growth rates of the batch cultures. Specific growth rate, μ in d^{-1}, is the slope of the regression of ln-transformed cell densities (Figure S-3).

culture condition	specific growth rate, μ [d^{-1}]	R^2 †
C1	0.48	0.994
C2	0.46	0.992
C3	0.43	0.998
C4	0.42	0.997
C5	0.43	0.998
C6	0.5	0.999

†For all culture conditions, p < 0.001.

Table S-4:
Settings of the NanoSIMS 50L instrument during acquisition of data from different samples.

**Measurement session**	**Aug 2016**	**Nov & Dec 2016**	**Jun 2018**	**Jan 2019**
Sample ID†	BS–2, EM–2, WM–10	EM–6, WM–1, WM–7, WM–9	C1–C6	C1, C2, C6, BS–2
**Pre-sputtering conditions**				
Primary ion beam current (in FC_o)	20 pA (in D1–3)	10 pA (in D1–3)	20 pA (in D1–3)	20 pA (in D1–3)
Diaphragm and slits	D1–1	D1–1	D1–1	D1–1
FOV size [μm×μm]	10 × 10 to 30 × 30	10 × 10 to 30 × 30	60 × 60	60 × 60
Duration [min]	1	2	2–3	5
**Image acquisition conditions**				
Primary ion beam current (in FC_o)	20 pA	10 pA	5 pA	10 pA
Diaphragm and slits	D1–3, ES–2	D1–3, ES–2	D1–3, ES–2	D1–3, ES–2
Detected secondary ions	$^{23}Na^+$, $^{24}Mg^+$, $^{44}Ca^+$, $^{88}Sr^+$	$^{23}Na^+$, $^{24}Mg^+$, $^{44}Ca^+$, $^{88}Sr^+$	$^{23}Na^+$, $^{24}Mg^+$, $^{39}K^+$, $^{44}Ca^+$, $^{88}Sr^+$, $^{138}Ba^+$	$^{23}Na^+$, $^{24}Mg^+$, $^{39}K^+$, $^{44}Ca^+$, $^{88}Sr^+$, $^{138}Ba^+$
Dwell time [μs pixel^{-1}]	1000	1000	1000	1000
FOV size [μm×μm]	7 × 7 to 20 × 20	5 × 5 to 10 × 10	6 × 6 to 13 × 13	6 × 6 to 13 × 13
Image size [pixel × pixel]	128 × 128, occasionally 64 × 64 and 256 × 256	64 × 64, occasionally 128 × 128	64 × 64, occasionally 128 × 128	64 × 64
Number of planes	1000	1000	1000	1000

†Abbreviations of the field samples (BS = Black Sea, EM = Eastern Mediterranean, WM = Western Mediterranean, sampling station numbers shown in Figure 6) and cultured samples (C, culturing conditions shown in Supplementary Figure S-1 and Table S-2).
